# Molecular epidemiology and genomic evolution mechanism of carbapenemase-resistant *Enterobacter cloacae complex* in Northeast China from July 2019 to March 2025

**DOI:** 10.3389/fmicb.2026.1767690

**Published:** 2026-03-23

**Authors:** Ze Li, Chun Yang, Jia Du, Heyuan Guan, Jiaxin Wang, Qi Sun, Jiancheng Xu

**Affiliations:** Department of Laboratory Medicine, Center of Infectious Diseases and Pathogen Biology, First Hospital of Jilin University, Changchun, China

**Keywords:** *bla*_KPC_, *bla*_NDM_, carbapenemases, *Enterobacter cloacae complex*, IncX3, whole genome sequencing

## Abstract

The rise of carbapenem-resistant Enterobacteriaceae (CRE), particularly the *Enterobacter cloacae complex* (ECC), poses a significant global health threat. However, the genomic evolution and transmission mechanisms of CRECC remain insufficiently understood. This study aimed to characterize the genotypes, resistance determinants, and transmission dynamics of clinical CRECC isolates using whole-genome sequencing (WGS) to inform infection control strategies. A total of 123 clinical CRECC isolates were subjected to species identification, antimicrobial susceptibility testing, WGS, plasmid analysis, and conjugation experiments. Taxonomic classification was refined based on an Average Nucleotide Identity (ANI) threshold of ≥95%, and phenotypic confirmation of carbapenemase production was performed using the combined disc test. *Enterobacter hormaechei* was the predominant species (*n* = 114, 92.7%), comprising three major subspecies: *xiangfangensis, steigerwaltii, and hoffmannii*. ST171 (30.1%) and ST1120 (21.1%) emerged as the dominant clonal lineages. The primary resistance genes were *bla*_NDM − 5_ (55.3%) and *bla*_NDM − 1_ (39.0%), with a subset of isolates co-harboring *bla*_KPC − 2_. All isolates were resistant to meropenem and imipenem (MIC_90_ ≥16 μg/ml). Genomic analysis revealed that the IncX3 plasmid was the principal vector for *bla*_NDM_, facilitating its dissemination. Minimal SNP differences between isolates within the same clonal group suggested recent transmission events. Conjugation experiments confirmed the horizontal transferability of *bla*_NDM_-carrying plasmids, with a success rate of 28.0% (7/25). This study reveals that the regional epidemiology of CRECC is driven by the clonal expansion of *E. hormaechei* ST171 and ST1120, coupled with the efficient horizontal transfer of *bla*_NDM_ genes via IncX3 plasmids. These findings provide a crucial molecular basis for targeted surveillance and infection control measures to mitigate the spread of high-risk CRECC clones.

## 1 Introduction

Antimicrobial Resistance (AMR) represents a paramount global health challenge, seriously compromising the efficacy of infection treatment (Rahman et al., 2026). The rapid dissemination of carbapenem-resistant Enterobacteriaceae (CRE) is particularly alarming. As carbapenems serve as the last line of defense against multidrug-resistant Gram-negative infections, their compromised efficacy has placed clinicians in an increasingly precarious therapeutic landscape. Among the numerous CRE pathogens, the *Enterobacter cloacae complex* (ECC) is a significant opportunistic human pathogen whose clinical relevance is frequently underestimated. Unlike the more extensively studied *Klebsiella pneumoniae* and *Escherichia coli*, ECC is not a single species but a heterogeneous group comprising over 10 distinct genetic clusters (Candela et al., 2023). This complex taxonomy, combined with high genomic plasticity, positions ECC as a reservoir for antimicrobial resistance genes—particularly carbapenemase genes (e.g., *bla*_KPC_, *bla*_NDM_, *bla*_IMP_) (Suzuki et al., 2024; Falco et al., 2021; Kovarova et al., 2025). Recent clinical surveillance data indicate a global rise in the isolation rate of carbapenemase-producing *Enterobacter cloacae complex* (CRECC). These pathogens frequently cause severe nosocomial infections, including bloodstream infections, ventilator-associated pneumonia, and complicated urinary tract infections, which are associated with high mortality rates (Denissen et al., 2022; Fata et al., 1996). Although existing studies have preliminarily characterized the resistance phenotypes and molecular epidemiology of CRECC, significant knowledge gaps remain regarding the underlying mechanisms of its genomic evolution. Given the diversity of sub-types within CRECC, the associations between distinct sequence types (STs) and specific carbapenemase genes lack systematic analysis at the whole-genome level. This study aims to conduct in-depth genotype and evolutionary analyses of clinically isolated CRECC using whole-genome sequencing (WGS). By constructing high-resolution phylogenetic trees and resistance gene transmission networks, we seek to elucidate the evolutionary trajectories and transmission mechanisms of CRECC at the molecular level. This approach will identify potential high-risk clones and provide a solid theoretical foundation for formulating precise clinical infection control strategies and antibiotic stewardship plans.

## 2 Materials and methods

### 2.1 Strain collection and preservation

A total of 123 carbapenem-resistant bacterial isolates were recovered from clinical specimens at the First Hospital of Jilin University, Jilin Province, China, between July 2019 and March 2025. The sources of these specimens included ascites (*n* = 2), bronchoalveolar lavage fluid (*n* = 1), bile (*n* = 1), blood (*n* = 26), synovial fluid (*n* = 1), pleural effusion (*n* = 2), pus (*n* = 2), cerebrospinal fluid (*n* = 3), sputum (*n* = 54), tissue (*n* = 1), and urine (*n* = 30). For long-term preservation, the isolates were suspended in 50% glycerol at a 1:1 ratio (yielding a final concentration of 25%) and stored in a −80 °C freezer. This study was approved by the Ethics Committee of the First Hospital of Jilin University (Approval No. 2025-MS-268), and all sample processing was conducted in strict compliance with patient privacy protection regulations.

### 2.2 Specimen recovery and bacterial cultivation

Frozen isolates were retrieved from the −80 °C freezer and allowed to thaw at room temperature for approximately 2 h. All subsequent manipulations were performed within a biosafety cabinet. An inoculation loop was used to transfer an aliquot of the bacterial suspension onto MacConkey agar plates using the three-zone streak method. The plates were properly labeled and incubated overnight at 37 °C in a constant-temperature CO_2_ incubator.

### 2.3 Antimicrobial susceptibility testing and genomic analysis

The panel of antimicrobial agents tested in this study included meropenem, imipenem, ceftriaxone, ceftazidime, ceftazidime/avibactam, tigecycline, polymyxin B, and amikacin. Antimicrobial susceptibility testing and result interpretation were performed in strict accordance with the Clinical and Laboratory Standards Institute (CLSI) M100 guidelines (2020 edition). Minimum Inhibitory Concentrations (MICs) for meropenem, imipenem, and polymyxin B were determined using the broth microdilution (BMD) method, while the susceptibility of other agents was evaluated using the Kirby-Bauer disk diffusion method. Bacterial colonies cultured at 35 °C for 16–18 h were suspended in sterile saline and adjusted to a turbidity of 0.5 McFarland before being evenly inoculated onto Mueller-Hinton (M-H) agar plates. Antibiotic discs were dispensed using sterile forceps, and inhibition zone diameters were measured using a caliper. Carbapenem resistance was defined as a meropenem or imipenem MIC ≥ 4 μg/ml. For genomic analysis, a single colony was inoculated into 3 ml of Luria–Bertani (LB) broth and incubated at 37 °C with shaking at 220 rpm for 24 h. A 1.5 ml aliquot of the culture was centrifuged at 12,000 rpm for 5 min; the supernatant was discarded, and the pellet was retained for DNA extraction. Qualified DNA samples were fragmented to a size of approximately 350 bp using a Covaris ultrasonicator. Sequencing libraries were constructed through end-repair, A-tailing, adapter ligation, purification, and polymerase chain reaction (PCR) amplification. Library concentration was preliminarily quantified using Qubit 2.0 and diluted to 2 ng/μL. Insert sizes were verified using an Agilent 2100 Bioanalyzer. The effective library concentration was accurately quantified via qPCR to ensure quality. Qualified libraries were pooled based on effective concentration and data output requirements, then sequenced on the Illumina NovaSeq platform. The average sequencing depth for all isolates was 200 × , with genome coverage reaching 95%. Clean data for each isolate were assembled using SPAdes v3.15.1, and contigs shorter than 500 bp were discarded prior to evaluation and statistical analysis. For core-genome SNP (cgSNP) analysis, sequences were aligned against the reference genome [NZ_CP077308.1] using Snippy v4.6.0 (Torsten Seemann, Australia). To ensure the accuracy of phylogenetic inference, recombinant regions were identified and filtered using Gubbins v3.0.

Assembled genomic sequences were annotated against the NCBI non-redundant protein (NR) database. During this process, predicted protein sequences derived from the assemblies were aligned with protein sequences of known species in the NR database using BLASTp. Taxonomic identification was refined using FastANI v1.34. Pairwise ANI values were calculated between the query genomes and reference type strains (*E. hormaechei subsp. steigerwaltii, subsp. xiangfangensis, and subsp. hoffmannii*). An ANI cut-off score of ≥95% was applied to define species and subspecies boundaries. Plasmid identification and typing were performed using PlasmidFinder (https://cge.cbs.dtu.dk/services/PlasmidFinder) (Gschwind et al., 2023) and plasmid Multilocus Sequence Typing (pMLST, https://cge.cbs.dtu.dk/services/pMLST/) (Zhou et al., 2022). Antimicrobial resistance genes were identified using ResFinder (https://cge.cbs.dtu.dk/services/ResFinder) (Bortolaia et al., 2020), with results supplemented by a colloidal gold immunochromatographic assay for carbapenemase detection. Contigs harboring the *bla*_NDM_ gene were extracted using SeqKit v0.8.0, and their size and GC content were quantified (Shen et al., 2016). Pairwise Single Nucleotide Polymorphism (SNP) comparisons were conducted using R software (version 4.3.3) (R Foundation for Statistical Computing, Vienna, Austria). Finally, the phylogenetic trees were annotated and visualized using the Interactive Tree of Life (iTOL) online tool (https://itol.embl.de/).

### 2.4 Conjugation transfer experiments

A subset of 25 isolates was randomly selected from the initial 123 strains to undergo conjugation experiments using the filter mating method (Bi et al., 2018). In these assays, isolates harboring carbapenem resistance genes (CRGs) served as donors, while the rifampicin-resistant *E. coli* strain EC600 served as the recipient. Transconjugants were selected by inoculating the mating mixtures onto Luria–Bertani (LB) agar plates supplemented with 30 μg/ml rifampicin and 1 μg/ml carbapenem. Putative transconjugants were subsequently verified using MALDI-TOF/MS and polymerase chain reaction (PCR). A colony was confirmed as a transconjugant if it carried the specific carbapenemase gene and exhibited Minimum Inhibitory Concentrations (MICs) for carbapenems and cephalosporins (determined by broth microdilution) exceeding those of the recipient EC600 strain.

### 2.5 PBA and EDTA synergistic inhibition assays

Carbapenemase production was phenotypically verified using the combined disc test. Bacterial colonies were suspended in sterile saline to achieve a turbidity equivalent to a 0.5 McFarland standard and inoculated onto Mueller-Hinton (M-H) agar plates to form a confluent lawn. Three meropenem (MEM, 10 μg) discs were placed on each inoculated plate. To inhibit specific carbapenemases, 10 μl of phenylboronic acid (PBA, 40 mg/ml) solution and 10 μl of EDTA (0.1 M) solution were added to the first and second MEM discs, respectively. The third MEM disc served as a growth control (meropenem alone). The discs were positioned at appropriate distances to prevent zone overlapping. Plates were incubated at 35 °C for 16–18 h. A positive result for carbapenemase production (KPC or Metallo-β-lactamase) was defined as an expansion of the inhibition zone diameter by ≥5 mm around the inhibitor-supplemented disc compared to the meropenem-only control disc. Resistance phenotypes were classified according to zone diameters: ≤ 19 mm indicated resistance (R), and ≥23 mm indicated susceptibility (S) (Chew et al., 2025).

### 2.6 Statistic analysis

Statistical analyses were performed using SPSS software, version 22.0 (IBM Corporation, Armonk, NY, USA). Continuous variables were expressed as mean ± standard deviation or median (interquartile range), as appropriate. Categorical variables, such as the prevalence of plasmid replicons across different specimen types and the distribution of resistance genes among different sequence types, were described as frequencies and percentages. Comparisons between groups (e.g., plasmid prevalence in urine vs. sputum) were evaluated using the Chi-square (χ^2^) test or Fisher's exact test for small sample sizes (*n* < 5). A two-tailed *P* < 0.05 was considered statistically significant.

## 3 Results

### 3.1 Species identification and genotypic distribution

Refined taxonomic identification based on ANI revealed that the majority of the 123 carbapenem-resistant isolates belonged to the *E. hormaechei* lineage (*n* = 114, 92.7%). Further subspecies-level discrimination classified these isolates into *E. hormaechei subsp. xiangfangensis* (*n* = 59, 48.0%), *E. hormaechei subsp. steigerwaltii* (*n* = 36, 29.3%), and *E. hormaechei subsp. hoffmannii* (*n* = 19, 15.4%). The remaining nine isolates (7.3%) were identified as other species within the ECC, based on an ANI threshold of ≥95% with type strains.

MLST identified 35 distinct sequence types (STs), with ST171 (*n* = 37, 30.1%) and ST1120 (*n* = 26, 21.1%) emerging as the predominant clonal lineages. Notably, ST171 was primarily associated with *E. hormaechei subsp. steigerwaltii*, while ST1120 was more common among other subspecies. Regarding carbapenemase determinants, *bla*_NDM − 5_ exhibited the highest prevalence (68/123, 55.3%, including dual-producers), followed by *bla*_NDM − 1_ (48/123, 39.0%).

Temporal analysis from July 2019 to March 2025 revealed a distinct clonal transition within our center. ST1120 was the dominant lineage during the early study period (2019–2021), representing 60.0% (18/30) of the isolates. However, a significant shift occurred starting in 2022, with the high-risk clone ST171 emerging as the most prevalent sequence type, accounting for 36.3% (33/91) of the isolates collected between 2022 and 2025. Correspondingly, while *bla*_NDM − 5_ was initially the primary carbapenemase gene, the frequency of *bla*_NDM − 1_ increased sharply from 2023 onwards. Furthermore, a stratified analysis showed that 28.5% (35/123) of the isolates were recovered from invasive sources (sterile sites such as blood and CSF), while 71.5% (88/123) were from non-invasive sources (sputum, urine, and pus). Notably, ST1120 was more frequently associated with invasive infections (40.0%, 14/35), whereas ST171 was the predominant clone among non-invasive respiratory and urinary tract isolates (34.1%, 30/88).

**Figure 1:**
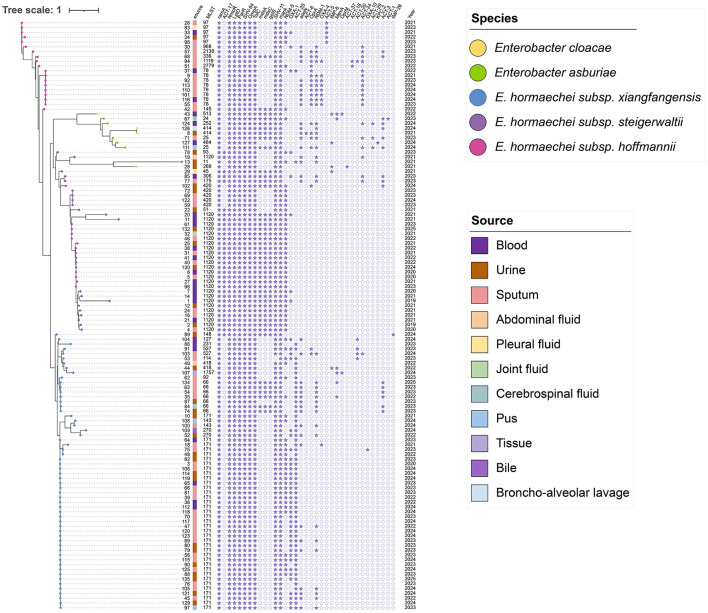
Phylogenetic relationships and resistance gene distribution map of CRECC isolates (2019–2025).

The distribution and prevalence of distinct MLST types across the CRECC isolates are illustrated in Figure 1.

### 3.2 Antimicrobial susceptibility profiles

The results of *in vitro* antimicrobial susceptibility testing demonstrated that all isolates were resistant to meropenem and imipenem (MIC ≥ 4 μg/ml). Overall, these strains exhibited high-level carbapenem resistance, with MIC_50_ and MIC_90_ values for both agents reaching or exceeding the upper detection limit (≥16 μg/ml). Additionally, the isolates were uniformly resistant to the third-generation cephalosporins ceftriaxone and ceftazidime (100% resistance). Attributable to the high prevalence of MBL (specifically NDM), the resistance rate to the novel inhibitor combination ceftazidime-avibactam was also notably high (92.3%−96.7%). In contrast, the isolates retained relatively high susceptibility to polymyxin B (resistance rate: 7.7%−15.4%), while displaying moderate resistance to tigecycline (38.5%−46.3%) and amikacin (46.2%−57.7%; Table 1).

**Table 1:** Antimicrobial susceptibility profiles of the bacterial isolates.

**Anti- -microbial Agent**	**Sputum (*****n*** = **54)**				**Urine (*****n*** = **30)**				**Blood (*****n*** = **26)**				**Others (*****n*** = **13)**			
	**MIC range (**μ**g/ml)**	**MIC**_50_	**MIC**_90_	**Resistance**, ***n*** **(%)**	**MIC range (**μ**g/ml)**	**MIC**_50_	**MIC**_90_	**Resistance**, ***n*** **(%)**	**MIC range (**μ**g/ml)**	**MIC**_50_	**MIC**_90_	**Resistance**, ***n*** **(%)**	**MIC range (**μ**g/ml)**	**MIC**_50_	**MIC**_90_	**Resistance**, ***n*** **(%)**
Meropenem	4–≥16	≥16	≥16	54 (100)	8–≥16	≥16	≥16	30 (100)	8–≥16	≥16	≥16	26 (100)	4–≥16	≥16	≥16	13 (100)
Imipenem	4–≥16	≥16	≥16	54 (100)	8–≥16	≥16	≥16	30 (100)	4–≥16	≥16	≥16	26 (100)	8–≥16	≥16	≥16	13 (100)
Ceftriaxone	64–≥256	≥256	≥256	54 (100)	128–≥256	≥256	≥256	30 (100)	64–≥256	≥256	≥256	26 (100)	32–≥256	128	≥256	13 (100)
Ceftazidime	32–≥256	≥256	≥256	54 (100)	64–≥256	≥256	≥256	30 (100)	64–≥256	≥256	≥256	26 (100)	32–≥256	≥256	≥256	13 (100)
Ceftazidime/ avibactam	8–≥256	128	≥256	51 (94.4)	16–≥256	≥256	≥256	29 (96.7)	16–≥256	128	≥256	25 (96.2)	8–≥256	64	≥256	12 (92.3)
Tigecycline	0.5–16	4	8	25 (46.3)	1–8	2	8	13 (43.3)	0.5–16	4	8	12 (46.2)	1–8	2	4	5 (38.5)
PolymyxinB	0.25–8	1	4	7 (13.0)	0.5–4	1	2	3 (10.0%)	0.5–8	2	4	4 (15.4%)	0.25–2	0.5	2	1 (7.7%)
Amikacin	4–≥64	16	≥64	28 (51.9)	8–≥64	32	≥64	16 (53.3%)	4–≥64	32	≥64	15 (57.7%)	8–32	16	32	6 (46.2%)

All MIC values are expressed in μg/ml. Definitions: MIC_50_ and MIC_90_ represent the concentrations that inhibit 50% and 90% of the bacterial isolates, respectively. Others: This category includes isolates recovered from ascites (n = 2), bronchoalveolar lavage fluid (n = 1), bile (n = 1), synovial fluid (n = 1), pleural effusion (n = 2), pus (n = 2), cerebrospinal fluid (n = 3), and tissue (n = 1).

### 3.3 Plasmid analysis of the 123 CRECC isolates

Given that antimicrobial resistance genes (ARGs) are frequently harbored by mobile genetic elements such as plasmids, this study employed WGS to characterize plasmid replicon types in the 123 CRECC isolates (Supplementary Table S2). The analysis revealed that the isolates carried diverse incompatible (Inc) plasmids, predominantly IncX3 (69.9%), followed by IncHI2A (6.5%) and IncFII (6.5%). Notably, the IncX3 plasmid was primarily associated with the dissemination of *bla*_NDM − 5_.

Stratification by specimen type showed that IncX3 was highly prevalent in urine (24/30, 80.0%), blood (19/26, 73.1%), and sputum (38/54, 70.4%). Although the detection rate of IncX3 was numerically higher in urine compared to sputum, statistical analysis revealed no significant difference in plasmid distribution between these specimen types (*P* > 0.05). Similarly, no significant difference was observed for IncHI2A distribution between sputum (14.8%) and blood (3.8%; *P* = 0.25, Fisher's exact test). This uniform distribution suggests that the IncX3 plasmid possesses a broad host range, facilitating stable persistence across diverse human host niches regardless of the infection site. Co-occurrence analysis confirmed that 100% (68/68) of the *bla*_NDM − 5_-harboring isolates in this study also carried the IncX3 plasmid replicon, providing robust evidence for the stable association between this specific plasmid backbone and the dissemination of *bla*_NDM − 5_.

### 3.4 Genomic characteristics of carbapenemase-producing isolates

**Figure 2:**
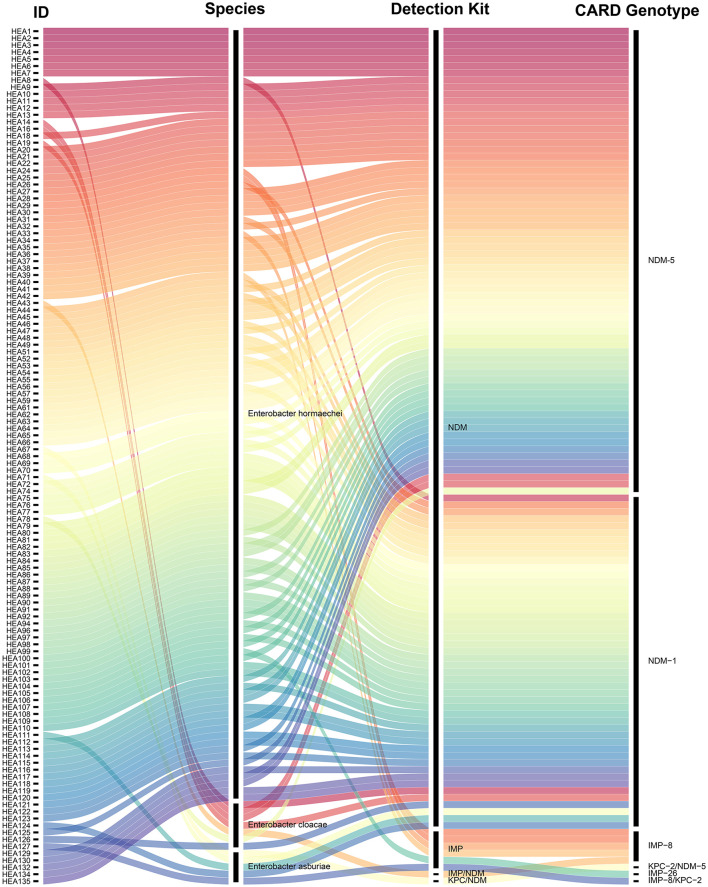
Sankey diagram illustrating the correlation between species taxonomy, phenotypic carbapenemase production, and genotypic resistance determinants among 123 CRECC isolates. The diagram visualizes the data flow from isolate identification (ID) and species classification to phenotypic detection via a colloidal gold immunochromatographic assay (Detection Kit) and genotypic profiling via Whole Genome Sequencing (CARD Genotype). The width of each flow is proportional to the number of isolates. Two isolates exhibited phenotypic-genotypic discrepancies: HEA43 was phenotypically positive for both *bla*_IMP_ and *bla*_NDM_ using the detection kit but was genotypically identified as carrying only the *bla*_IMP − 8_ gene. HEA127 was phenotypically positive for *bla*_IMP_ but was genotypically found to harbor both *bla*_IMP − 8_ and *bla*_KPC − 2_.

Analysis of the 123 genomic sequences identified two classes of CRGs: Class A serine carbapenemases (specifically *bla*_KPC_) and Class B MBLs (specifically *bla*_NDM_ and *bla*_IMP_). No Class D carbapenemases (*bla*_OXA_) were detected. All 123 CRECC isolates harbored carbapenemase-encoding genes. The majority (121 isolates) carried a single carbapenemase gene, including *bla*_NDM − 5_ (*n* = 67), *bla*_NDM − 1_ (*n* = 48), *bla*_IMP − 8_ (*n* = 5), and *bla*_IMP − 26_ (*n* = 1). Two isolates harbored dual carbapenemase genes: one carried both *bla*_IMP − 8_ and *bla*_KPC − 2_, while the other carried *bla*_KPC − 2_ and *bla*_NDM − 5_. Additionally, phenotypic carbapenemase confirmation tests were performed on all 123 isolates. With the exception of isolates HEA43 and HEA127, the phenotypic results for 121 isolates were consistent with the genotypic identification provided by ResFinder, yielding a concordance rate of 98.37% (Figure 2).

This study characterized the genomic data of all CRECC isolates harboring at least one copy of the *bla*_NDM_ gene. WGS analysis of the 116 *bla*_NDM_-positive isolates revealed that the *bla*_NDM_-harboring contigs ranged in length from 1,850 to 84,004 bp, with a GC content of 46.3%−64.2% (Supplementary Table S4). The *bla*_NDM_ gene was the predominant CRG among the 123 CRECC isolates (116/123, 94.3%), identified in specimens including sputum (*n* = 54), urine (*n* = 28), blood (*n* = 23), cerebrospinal fluid (*n* = 3), ascites (*n* = 2), pus (*n* = 2), synovial fluid (*n* = 1), pleural effusion (*n* = 1), bile (*n* = 1), and bronchoalveolar lavage fluid (*n* = 1). The *bla*_IMP_ gene represented the second most prevalent CRG (7/123, 5.7%), detected in blood (*n* = 3), urine (*n* = 2), pleural effusion (*n* = 1), and tissue (*n* = 1).

To investigate transmission dynamics, pairwise core genome single nucleotide polymorphism (cgSNP) analysis was performed on isolates sharing the same MLST type. This revealed the presence of highly homologous clusters within multiple clonal lineages (Supplementary Table S5). Notably, several isolate pairs exhibited minimal genetic divergence ( ≤ 4 SNPs), strongly suggesting origin from recent transmission events. For instance, a distance of only two SNPs was observed between pairs HEA36/HEA56 (ST171), HEA14/HEA40 (ST1120), and HEA110/HEA113 (ST78). Additionally, pairs separated by four SNPs were identified within ST171 (HEA79/HEA80, HEA39/HEA66) and ST1120 (HEA6/HEA12, HEA11/HEA40). These limited SNP distances provide robust genomic evidence of close clonal relatedness between these isolates.

### 3.5 PBA/EDTA co-experiment

**Figure 3:**
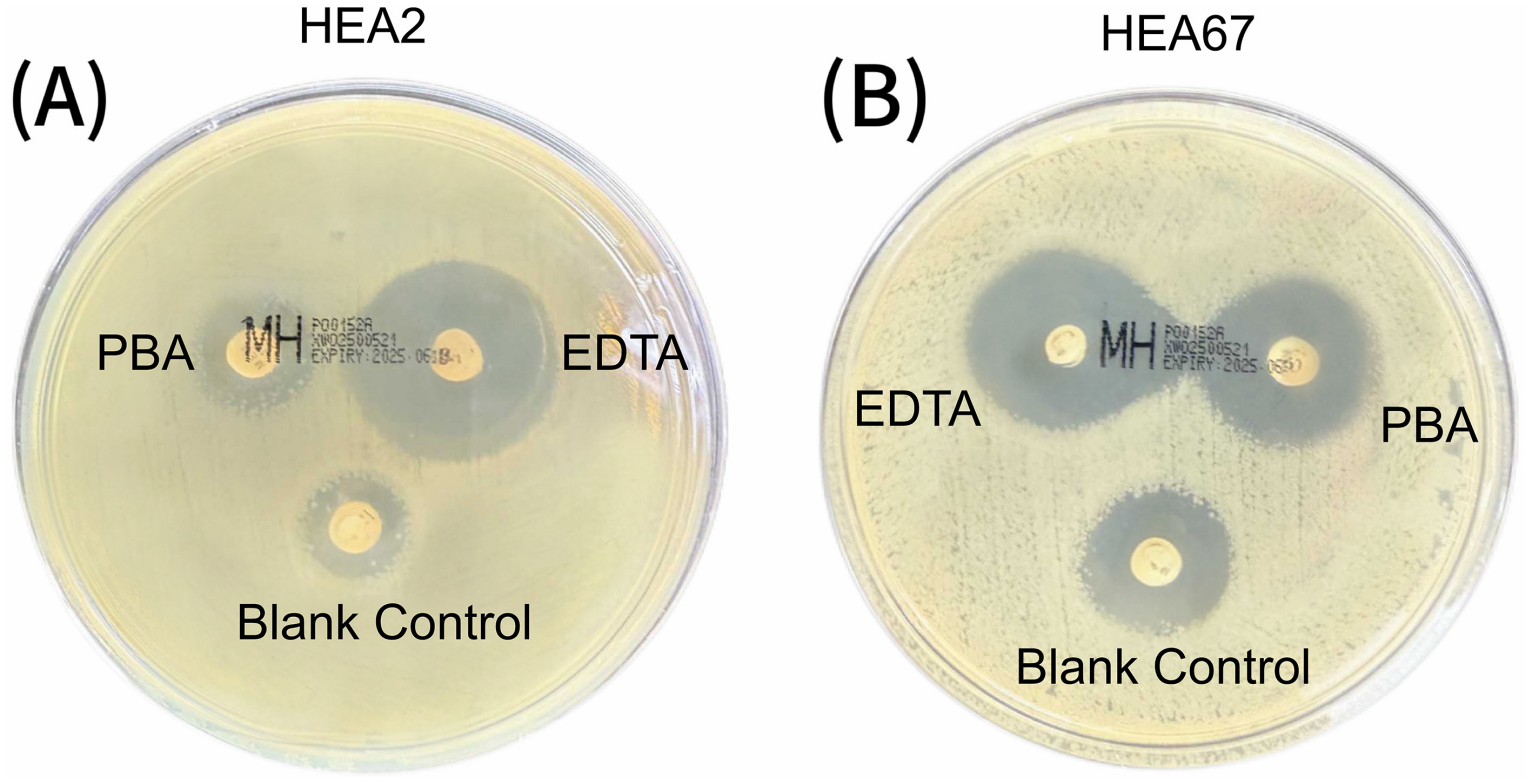
Phenotypic detection of carbapenemases using the combined disc test. **(A)** Isolate HEA2 (harboring *bla*_NDM − 5_). A significant expansion of the inhibition zone (≥5 mm) is observed around the disc supplemented with EDTA (Metallo-β-lactamase inhibitor) compared to MEM alone, indicating the production of MBLs. No synergistic effect is seen with PBA; **(B)** Isolate HEA67 (co-harboring *bla*_NDM − 5_ and *bla*_KPC − 2_). Significant inhibition zone expansions are observed around both the EDTA- and PBA-supplemented discs compared to MEM alone, confirming the simultaneous production of MBL and KPC carbapenemases.

The phenotypic detection results of the paper strip co-test are shown in Figure 3. Among the 123 isolates, which were all genotypically confirmed as Metallo-β-lactamase (MBL) producers (carrying *bla*_NDM_ or *bla*_IMP_, 122 isolates tested positive in the EDTA co-test, yielding a genotype-phenotype concordance rate of 99.2% (122/123). Only one isolate yielded a false-negative result in the EDTA test. regarding the detection of *bla*_KPC_, although the prevalence was low (*n* = 2), the PBA co-test successfully identified both positive isolates (2/2). Notably, these two isolates were dual-producers (co-harboring *bla*_KPC_ with *bla*_NDM_ or *bla*_IMP_), yet the presence of MBLs did not interfere with the accurate detection of KPC.

### 3.6 Transferability of CRGs

Twenty-five clinical CRECC isolates harboring CRGs were selected as donors for conjugation assays. Successful transfer was observed in seven cases, yielding an overall conjugation rate of 28.0% (7/25). MALDI-TOF/MS identification and subsequent sequencing confirmed the transfer of *bla*_NDM − 1_ (from isolates HEA30, HEA45, HEA68, HEA70, and HEA97) and *bla*_NDM − 5_ (from isolates HEA78 and HEA90). Antimicrobial susceptibility testing demonstrated that plasmid acquisition conferred high-level carbapenem resistance to the recipient strain. Compared to the recipient control *E. coli* EC600 (meropenem and imipenem MICs ≤ 1 μg/ml), all seven transconjugants exhibited significantly elevated MICs. Notably, transconjugants harboring *bla*_NDM − 5_ (derived from HEA78 and HEA90) displayed extreme resistance, with MICs for meropenem and imipenem rising to ≥32 μg/ml. Similarly, the five *bla*_NDM − 1_-carrying transconjugants showed meropenem MICs increasing to 16–64 μg/ml. These results indicate that the *bla*_NDM − 1_ and *bla*_NDM − 5_ genes identified in this study are located on conjugative plasmids capable of horizontal transfer, thereby posing a significant risk of further dissemination (Table 2).

**Table 2:** MICs of CRG-carrying isolates and their corresponding transconjugants.

**Strain ID**	**Type**	**Genotype**	**Meropenem**	**Imipenem**
EC600	Recipient	–	≤ 1	≤ 1
HEA30	Donor	*bla*_NDM − 1_	32	32
HEA30-TC	Transconjugant	*bla*_NDM − 1_	16	16
HEA45	Donor	*bla*_NDM − 1_	64	32
HEA45-TC	Transconjugant	*bla*_NDM − 1_	32	32
HEA68	Donor	*bla*_NDM − 1_	>64	64
HEA68-TC	Transconjugant	*bla*_NDM − 1_	32	32
HEA70	Donor	*bla*_NDM − 1_	32	32
HEA70-TC	Transconjugant	*bla*_NDM − 1_	16	16
HEA97	Donor	*bla*_NDM − 1_	16	16
HEA97-TC	Transconjugant	*bla*_NDM − 1_	16	8
HEA78	Donor	*bla*_NDM − 5_	>64	>64
HEA78-TC	Transconjugant	*bla*_NDM − 5_	64	64
HEA90	Donor	*bla*_NDM − 5_	>64	>64
HEA90-TC	Transconjugant	*bla*_NDM − 5_	32	32

## 4 Discussion

Carbapenems, serving as the “last line of defense” among β-lactam antibiotics, function by inhibiting penicillin-binding proteins (PBPs) to disrupt cell wall synthesis, thereby holding immense clinical value (Papp-Wallace et al., 2011). However, the global dissemination of CRE presents a severe public health challenge. Epidemiological data highlight significant regional variations in CRE resistance rates. For instance, in Uganda, resistance to ertapenem and imipenem in *E. coli* and *K. pneumoniae* is approximately 10%, whereas in Madagascar, imipenem resistance in *K. pneumoniae* reaches as high as 28.8% (Ma et al., 2023). These data underscore the high potential of CRE for transregional dissemination and the substantial threat it poses. Consequently, there is an urgent need for in-depth research into the clinical molecular epidemiology of CRECC in this specific region.

This study identified *E. hormaechei* as the predominant species causing CRECC infections in this region, a finding consistent with numerous previous studies. Prior research has indicated that *E. hormaechei subsp. hoffmannii* is the dominant clade within CRECC, followed by *subsp. steigerwaltii* and *subsp. xiangfangensis*, often accompanied by diverse STs such as ST78, ST93, and ST171. This reflects the high population structure diversity and clonal heterogeneity of this species (Yang et al., 2025; Cai et al., 2024). Our study not only confirmed the dominance of *E. hormaechei* but also identified ST171 and ST1120 as the prevailing clonal types locally. This suggests that *E. hormaechei* is not only a critical reservoir for CRECC but that specific high-risk clones (e.g., ST171) may play a pivotal role in the maintenance and dissemination of antimicrobial resistance, exhibiting significant heterogeneity and environmental adaptability.

In this study, the IncX3 plasmid was detected at a remarkably high prevalence among *bla*_NDM_-harboring isolates, and conjugation assays confirmed its ability to successfully transfer resistance to recipient strains. The IncX3 plasmid is a narrow-host-range plasmid primarily found in Enterobacteriaceae, characterized by high conjugative efficiency, stability, negligible fitness cost, and the ability to enhance biofilm formation in host bacteria (Guo et al., 2022). The carbapenemase gene *bla*_NDM_ is a core determinant mediating carbapenem resistance in Gram-negative bacteria. Plasmids serve as key vectors for the horizontal transmission of this gene, and their types and distribution directly influence the prevalence of resistant strains. Previous studies have noted that the plasmid localization of *bla*_NDM_ exhibits significant strain-specific and genotype-dependent clustering; for instance, in ST22 isolates of Citrobacter portucalensis (CPCF), all *bla*_NDM_-carrying plasmids were of the IncX3 type (Wang Q. et al., 2025). A retrospective analysis in China revealed that the backbone structure of IncX3 plasmids has remained highly conserved over the past decade (Liu et al., 2021). The accessory modules, primarily consisting of transposase genes, metabolic genes, and resistance determinants, are acquired DNA fragments integrated into the backbone via mobile genetic elements (Shi et al., 2018). The IncX3 plasmid demonstrates exceptional stability; it persists during continuous passage under selective antibiotic pressure (Flerlage et al., 2020) and shows no significant loss after 100 generations in clinical isolates or transconjugants (Tian et al., 2020). Similarly, an IncX3 *bla*_NDM − 5_ plasmid in a clinical *K. pneumoniae* isolate remained stable after 5 days of repeated subculturing (Zhu et al., 2020). These characteristics suggest that once introduced into a clinical setting, IncX3 plasmids can persist long-term within host bacteria or as a “silent reservoir” in the hospital environment, even without continuous antibiotic pressure (Wang X. et al., 2025). Consistent with these observations, our study found a strong association between the *bla*_NDM_ gene (particularly *bla*_NDM − 5_) and the IncX3 plasmid. This association significantly facilitates the horizontal dissemination of carbapenem resistance across different bacterial species, representing a critical molecular mechanism contributing to the worsening clinical resistance landscape.

To accurately identify MBLs, we employed a phenotypic detection method utilizing EDTA. The EDTA synergy test enhances the detection of metallo-enzymes, such as NDM, by inhibiting their activity via zinc ion chelation. Due to its simplicity, high sensitivity, and effectiveness, this method is widely applied in clinical microbiology laboratories (Liu et al., 2025). By comparing inhibition zones on standard Mueller-Hinton Agar (MHA) vs. MHA supplemented with inhibitors (EDTA/PBA) using meropenem, ertapenem, imipenem, and doripenem discs, the method effectively identifies MBL-producing Enterobacteriaceae (Birgy et al., 2012). In our study, MBL-producing isolates were successfully identified via the EDTA synergy test, yielding results highly consistent with genotypic detection. This reaffirms the practical value of this method for the rapid screening of metallo-enzymes like NDM, particularly in resource-limited settings.

Although this study elucidates the epidemiological characteristics and resistance mechanisms of clinical CRECC isolates in Jilin Province, several limitations warrant further exploration: (1) single-center design and sample size: the isolates were obtained exclusively from a single tertiary hospital. Due to this limited sample source and size, our study may have underestimated the diversity of resistance determinants, potentially missing low-frequency clones or rare resistance genes circulating in the community or other healthcare settings. Furthermore, while the First Hospital of Jilin University is a major medical center in the region, the findings primarily reflect the local prevalence within this specific clinical environment. Therefore, the generalizability of these results to the broader “Northeast China” region should be tempered, as resistance patterns and clonal distribution may vary significantly across different hospital tiers and geographic locations. Future multi-center studies with larger cohorts are required to provide a more comprehensive and representative view of the regional epidemiology. (2) Absence of animal models: this study relied on genomic and phenotypic analysis of clinical isolates without validating pathogenicity or virulence *in vivo*. Animal models are crucial for understanding pathogenic mechanisms and host adaptability. Future studies should incorporate animal experiments to evaluate virulence and transmissibility *in vivo*. (3) Biological factors underlying conjugation failures: although the IncX3 plasmid demonstrated efficient transfer, the overall conjugation success rate was 28% (7/25). However, comparative genomic analysis revealed that the *bla*_NDM_-carrying contigs in the non-conjugative isolates shared >99% nucleotide identity and synteny with the IncX3 plasmid backbones recovered from the successful transconjugants. The failure of plasmid transfer in the remaining *bla*_NDM − 5_-harboring isolates could be attributed to several biological barriers. Firstly, host restriction-modification (R-M) systems in either the donor or recipient (EC600) might trigger the degradation of incoming plasmid DNA. Secondly, plasmid incompatibility (Inc) groups may prevent the stable coexistence of the transferred plasmid within the host cell. Furthermore, the absence or truncation of essential transfer (tra) genes on the resistance plasmids could render them non-conjugative, requiring a helper plasmid for mobilization. Future research employing long-read sequencing could further elucidate the genetic architectures and specific mechanisms underlying these failures. (4) Lack of longitudinal transmission tracking: although samples were collected over time, we did not perform long-term tracking of nosocomial transmission chains. Understanding hospital-acquired infection routes and clonal evolution requires longitudinal surveillance. Future studies should implement long-term tracking (months to years) to better understand transmission dynamics and risks within the hospital environment. (5) Limitation of sequencing technology: our study relied on Illumina short-read sequencing, which restricted the ability to fully circularize and reconstruct complete plasmid sequences. Although we combined plasmid replicon typing, contig mapping, and conjugation experiments to rigorously verify the presence and transferability of resistance plasmids, the lack of long-read sequencing data prevented us from resolving the complete physical architectures of these plasmids. Future studies utilizing hybrid assembly approaches are warranted to obtain closed plasmid sequences and analyze their fine-scale structural variations.

In summary, this study systematically characterized the molecular epidemiology of CRECC at a major medical center in Northeast China. *E. hormaechei* (ST171/ST1120) was identified as the predominant epidemic clone, with the IncX3 plasmid serving as the primary vector for the *bla*_NDM_ gene and demonstrating efficient horizontal transfer capabilities. These findings enrich the epidemiological data on CRE in this region and provide a scientific basis for developing targeted infection control strategies, curbing the spread of resistant plasmids, and establishing regional resistance surveillance systems.

## 5 Conclusion

This study provides a comprehensive molecular epidemiological analysis of CRECC isolates from a major medical center in Northeast China. Our findings reveal a landscape dominated by high-risk clones of *E. hormaechei*, specifically ST171 and ST1120. The primary mechanism of resistance is the carriage of the *bla*_NDM_ gene, which is predominantly mobilized by the highly stable and transmissible IncX3 plasmid. Genomic evidence, supported by minimal SNP differences between isolates, points to active clonal transmission within the hospital setting. The successful horizontal transfer of these resistance plasmids was confirmed through conjugation experiments, underscoring the significant risk of further dissemination. These results provide a critical evidence base for implementing targeted surveillance, enhancing infection prevention and control protocols, and guiding antimicrobial stewardship efforts to mitigate the spread of these high-risk, multidrug-resistant pathogens.

## Data Availability

The datasets presented in this study can be found in online repositories. The names of the repository/repositories and accession number(s) can be found in the article/supplementary material.
